# High-quality draft genome sequence of nematocidal *Bacillus thuringiensis* Sbt003

**DOI:** 10.4056/sigs.4738557

**Published:** 2014-03-01

**Authors:** Yingying Liu, Weixing Ye, Jinshui Zheng, Lei Fang, Donghai Peng, Lifang Ruan, Ming Sun

**Affiliations:** State Key Laboratory of Agricultural Microbiology, College of Life Science and Technology, Huazhong Agricultural University, Wuhan, China.

**Keywords:** The Next-Generation sequencing, parasporal crystal protein, *Bacillus thuringiensis*

## Abstract

*Bacillus thuringiensis* represents one of the six species of "*Bacillus cereus* group" in the genus *Bacillus* within the family *Bacillaceae*. Strain Sbt003 was isolated from soil and identified as *B. thuringiensis*. It harbors at least seven plasmids and produces three shapes of parasporal crystals including oval, bipyramidal and rice. SDS-PAGE analysis of spore-crystal suspension of this strain reveals six major protein bands, which implies the presence of multiple parasporal crystal genes. Bioassay of this strain reveals that it shows specific activity against nematodes and human cancer cells. In this study, we report the whole genomic shotgun sequences of Sbt003. The high-quality draft of the genome is 6,175,670 bp long (including chromosome and plasmids) with 6,372 protein-coding and 80 RNA genes.

## Introduction

*Bacillus thuringiensis*, *B. cereus*, *B. anthracis* and other three species constitute the "*Bacillus cereus* group", a nontaxonomic term, within the genus *Bacillus* and family *Bacillaceae* [1]. These species were classified as separate species mainly based on their distinct phenotypes, although extensive genomic studies on strains of these species using different techniques have suggested that they form a single species [2-5]. Strain Sbt003 belongs to the species *B. thuringiensis*. The type strain of the species produces one or more parasporal crystal proteins showing specific activity against certain larvae from various orders of insects [6]. The specific role and the abundant number of genes encoding of insecticidal crystal proteins of this species have attracted much attention from both academic and industrial researchers. Dozens of *B. thuringiensis* strains have been sequenced, and dozens more are on their way. In this study, we present a summary classification and a set of features for *B. thuringiensis* Sbt003, together with the description of the genomic sequencing and annotation.

## Classification and features

**Figure 1:**
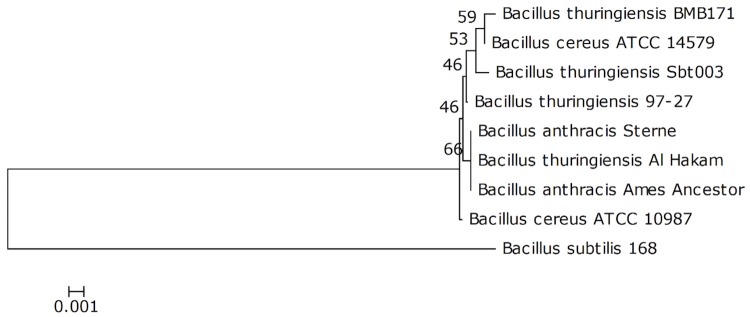
General characteristics of *Bacillus thuringiensis* Sbt003.(A) Agarose gel electrophoresis of total DNA of Sbt003. Lane M, molecular mass standard, Lambda DNA/*Hin*dIII; Lane 1, Sbt003. (B) Phase contrast micrograph of Sbt003 sporulated culture. (C) SDS-PAGE analysis of crystal proteins of Sbt003. Lane M, molecular mass standard; Lane 1, Sbt003.

*B. thuringiensis* strain Sbt003 harbors at least 7 plasmids and produces three different shapes of parasporal crystals including oval, bipyramidal and rice (Figure 1A, Figure 1B and Table 1). SDS-PAGE analysis of spore-crystal suspension of this strain reveals six major protein bands of 168.8, 148.5, 133.5, 117.2, 107.9 and 103.1 kDa, which implies the presence of multiple parasporal crystal genes (Figure 1C).

**Table 1:** Classification and general features of *B. thuringiensis* Sbt003 according to the MIGS recommendations [7]

**MIGS ID**	**Property**	**Term**	**Evidence code**^a^
		Domain *Bacteria*	TAS [8]
		Phylum *Firmicutes*	TAS [9-11]
		Class *Bacilli*	TAS [12,13]
	Current classification	Order *Bacillales*	TAS [14,15]
		Family *Bacillaceae*	TAS [14,16]
		Genus *Bacillus*	TAS [14,17,18]
		Species *Bacillus thuringiensis*	TAS [14,19]
		Type strain HD73	
	Gram stain	Gram-positive	NAS
	Cell shape	Rod-shaped	IDA
	Motility	Motile	NAS
	Sporulation	Spore-forming	IDA
	Temperature range	Room temperature	NAS
	Optimum temperature	28°C	IDS
	Carbon source	Organic carbon source	NAS
	Energy source	Organic carbon source	NAS
MIGS-6	Habitat	Soil	IDA
MIGS-6.3	Salinity	Salt tolerant	NAS
MIGS-22	Oxygen	Aerobic	NAS
MIGS-14	Pathogenicity	Avirulent	NAS
MIGS-4	Geographic location	Hubei, China	IDA
MIGS-4.1	Latitude	29-31N	
MIGS-4.2	Longitude	111-114E	
MIGS-4.3	Depth	5-10cm	
MIGS-4.4	Altitude	About 35m	
MIGS-5	Sample collection time	2000	IDA

a) Evidence codes - IDA: Inferred from Direct Assay; TAS: Traceable Author Statement (i.e., a direct report exists in the literature); NAS: Non-traceable Author Statement (i.e., not directly observed for the living, isolated sample, but based on a generally accepted property for the species, or anecdotal evidence). These evidence codes are from the Gene Ontology project [20].

**Figure 2:**
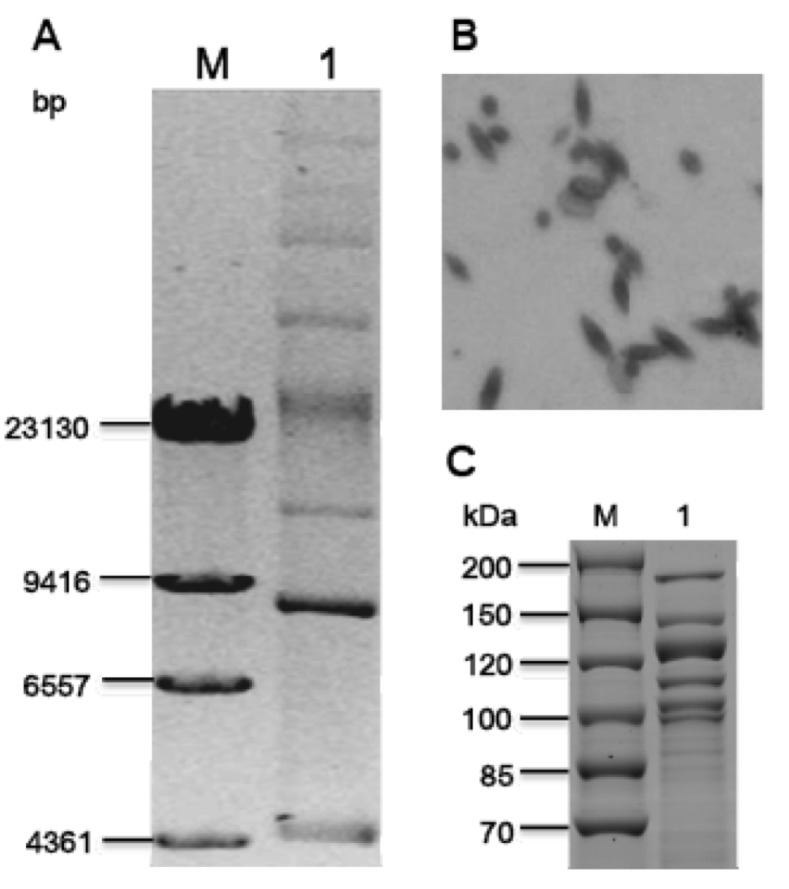
Neighbor-joining phylogenetic tree generated using MEGA 4 based on 16S rRNA sequences. The strains and their corresponding GenBank accession numbers (and, when applicable, draft sequence coordinates) for 16S rDNA sequences are: A, *B. thuringiensis* serovar *konkukian* str. 97-27 (AE017355.1): 9337-10763; B, *B. thuringiensis* BMB171 (CP001903): 9217-10643; C, *B. subtilis subsp. subtilis* str. 168 (NC_000964): 9839-11263; D, *B. cereus* ATCC 10987 (NC_003909): 9335-10761; E, *B. anthracis* str. 'Ames Ancestor' (NC_007530): 9335-10761; F, *B. anthracis* str. Sterne (NC_005945): 9336-10762; G, *B. thuringiensis* str. Al Hakam (NC_008600): 9336-10762; H, *B. cereus* ATCC 14579 (NC_004722): 28956-30382.

A representative genomic 16S rDNA sequence of strain Sbt003 was searched against GenBank database using BLAST [21]. Sequences showing more than 97% identity to the 16S rDNA of Sbt003 were selected for phylogentic analysis, and a 16S rDNA sequence from *B. subtilis subsp. subtilis* str. 168 was used as the outgroup. Nine sequences were aligned with ClustalW algorithm. The tree was reconstructed using neighbor joining with the Kimura 2-parameter substitution model. The phylogenetic tree was assessed by bootstrapping 1,000 times, and the consensus tree is shown in Figure 2.

## Genome sequencing and annotation

### Genome project history

This organism was selected for sequencing due to its specific activity against nematodes and human cancer cells. The complete high quality draft genome sequence is deposited in GenBank. The Beijing Genomics Institute (BGI) performed the sequencing and NCBI staff used the Prokaryotic Genome Automatic Annotation Pipeline (PGAAP) to complete the annotation. A summary of the project is given in Table 2.

**Table 2:** Genome sequencing project information

MIGS ID	Property	Term
MIGS-31	Finishing quality	High Quality Draft
MIGD-28	Libraries used	Two genomic libraries, one Illumina paired-end library (500 bp inserted size); one Illumina mate-pair library (2 kb inserted size)
MIGS-29	Sequencing platform	Illumina Hiseq 2000
MIGS-31.2	Sequencing coverage	488 ×
MIGS-30	Assemblers	SOAPdenovo 1.05 version
MIGS-32	Gene calling method	Glimmer and GeneMark
	GenBank Data of Release	Pending
	NCBI project ID	175950
	Project relevance	Biotechnological

### Growth conditions and DNA isolation

*B. thuringiensis* Sbt003 was grown in 50 mL Luria broth for 6 hours at 28°C. DNA was isolated by incubating the cells with lysozyme (10 mg/mL) in 2 mL TE (50 mM Tris base, 10 mM EDTA, 20% sucrose, pH8.0) at 4°C for 6 hours. 4 mL 2% SDS was added and the mixture was incubated at 55°C for 30 min; 2 mL 5M NaCl were added, and the mixture was incubated at 4°C for 10 min. DNA was purified by organic extraction and ethanol precipitation.

### Genome sequencing and assembly

The genome of *B. thuringiensis* Sbt003 was sequenced using Illumina Hiseq 2,000 platform (with a combination of a 100-bp paired-end reads sequencing from a 500-bp genomic library and a 90-bp mate-paired reads sequencing from a 2-kb genomic library). Reads with average quality scores below Q30 or having more than 3 unidentified nucleotides were eliminated. Using SOAPdenovo 1.05 version, 22,295,588 paired-end reads (achieving ~325 fold coverage [2.01 Gb]) and 11,166,312 mate-paired reads (achieving ~ 163 fold coverage [1.00 Gb]) were assembled *de novo* [22]. The assembly is considered a *high-guality draft* and consists of 104 contigs arranged in 61 scaffolds with a total size of 6,175,670 bp. According to bioinformatic analysis, we identified two large plasmids belonging to *ori44*-type and *repB*-type plasmids, respectively. The former plasmid has two *ori44*-type replicons. We propose it represents a fusion of two plasmids and its estimated size is about 200 kb. The latter plasmid has an expected size of at least 90 kb, according to the sequence of contig0027, which is typical of *repB*-type plasmids (80 ~ 90 kb). In addition, we identified five other plasmids from the plasmid pattern (see Figure 1A). The expected sizes of the smaller three are 13 kb, 8kb and 4kb, respectively, while the sizes of the larger two can't be deduced either from the plasmid pattern or by bioinformatic analysis.

### Genome annotation

Genome annotation was completed using the Prokaryotic Genomes Automatic Annotation Pipeline (PGAAP). Briefly, protein-coding genes were predicted using a combination of GeneMark and Glimmer [23-25]. Ribosomal RNAs were predicted by sequence similarity searching using BLAST against an RNA sequence database and/or using Infernal and Rfam models [26,27]. Transfer RNAs were predicted using tRNAscan-SE [28]. In order to detect missing genes, a complete six-frame translation of the nucleotide sequence was done and predicted proteins (generated above) were masked. All predictions were then searched using BLAST against all proteins from complete microbial genomes. Annotation was based on comparison to protein clusters and on the BLAST results. Conserved domain Database and Cluster of Orthologous Group information were then added to the annotation.

## Genome Properties

The high-quality draft assembly of the genome consists of 104 contigs in 61 scaffolds, with an overall 35.21% G+C content. Of the 6,452 genes predicted, 6,372 were protein-coding genes, and 80 RNAs were also identified. The majority of the protein-coding genes (66.67%) were assigned a putative function while the remaining ones were annotated as hypothetical proteins (Table 3). The distribution of genes into COGs functional categories is presented in Table 4.

**Table 3:** Genome Statistics

**Attribute**	**Value**	**% of total**
Genome size (bp)	6,175,670	100.00
DNA coding region (bp)	4,818,828	78.03
DNA G+C content (bp)	2,174,469	35.21
Number of scaffolds	61	-
Extrachromosomal elements	> 300 kb	> 4.86
Total genes	6,452	100.00
tRNA genes	70	1.08
rRNA genes	10	0.16
rRNA operons	0**	-
Protein-coding genes	6,372	98.76
Pseudo gene (Partial genes)	0 (49)	0 (0.76%)
Genes with function prediction (proteins)	4248	66.67%
Genes assigned to COGs	4,334	68.02%
Genes with signal peptides	437	6.86
CRISPR repeats	0	0

**none of the rRNA operons appears to be complete due to unresolved assembly problems.

**Table 4:** Number of genes associated with the general COG functional categories

Code	Value	% age	Description
J	224	4.404	Translation, ribosomal structure and biogenesis
A	0	0.0	RNA processing and modification
K	485	9.536	Transcription
L	374	7.354	Replication, recombination and repair
B	1	0.020	Chromatin structure and dynamics
D	48	0.944	Cell cycle control, cell division, chromosome partitioning
Y	0	0	Nuclear structure
V	143	2.812	Defense mechanisms
T	225	4.424	Signal transduction mechanisms
M	254	4.994	Cell wall/membrane/envelope biogenesis
N	59	1.160	Cell motility
Z	1	0.020	Cytoskeleton
W	1	0.020	Extracellular structures
U	65	1.278	Intracellular trafficking, secretion, and vesicular transport
O	122	2.399	Posttranslational modification, protein turnover, chaperones
C	215	4.227	Energy production and conversion
G	310	6.095	Carbohydrate transport and metabolism
E	480	9.438	Amino acid transport and metabolism
F	109	2.143	Nucleotide transport and metabolism
H	156	3.067	Coenzyme transport and metabolism
I	140	2.753	Lipid transport and metabolism
P	309	6.076	Inorganic ion transport and metabolism
Q	124	2.438	Secondary metabolites biosynthesis, transport and catabolism
R	783	15.395	General function prediction only
S	458	9.005	Function unknown
	2038	31.98	Not in COGs

The whole genomic sequence and the coding sequence of Sbt003 were analyzed by BtToxin_scanner [29], and eight potential crystal protein sequences were identified. Among these, four were considered to be full-length (locus tags: C797_02099, C797_12066, C797_12568 and C797_27783) while the others were considered to be truncated (Locus tags: C797_02094, C797_12046, C797_12061, C797_18417).
